# How Modifiable Are Modifiable Dementia Risk Factors? A Framework for Considering the Modifiability of Dementia Risk Factors

**DOI:** 10.14283/jpad.2023.119

**Published:** 2023-10-04

**Authors:** Lisa Bransby, E. Rosenich, P. Maruff, Y. Y. Lim

**Affiliations:** 1 Turner Institute for Brain and Mental Health, School of Psychological Sciences, Monash University, 18 Innovation Walk, Clayton, Melbourne, Victoria 3800 Australia; 2 Florey Institute of Neuroscience and Mental Health, Parkville, Victoria Australia; 3 Cogstate Ltd., Melbourne, Victoria Australia

**Keywords:** Dementia, risk factors, modifiable, prevention, risk reduction, Alzheimer’s disease

## Abstract

Many risk factors for dementia, identified from observational studies, are potentially modifiable. This raises the possibility that targeting key modifiable dementia risk factors may reduce the prevalence of dementia, which has led to the development of dementia risk reduction and prevention strategies, such as intervention trials or dementia prevention guidelines. However, what has rarely been considered in the studies that inform these strategies is the extent to which modifiable dementia risk factors can (1) be identified by individuals, and (2) be readily modified by individuals. Characteristics of modifiable dementia risk factors such as readiness of identification and targeting, as well as when they should be targeted, can influence the design, or success of strategies for reducing dementia risk. This review aims to develop a framework for classifying the degree of modifiability of dementia risk factors for research studies. The extent to which these modifiable dementia risk factors could be modified by an individual seeking to reduce their dementia risk is determined, as well as the resources that might be needed for both risk factor identification and modification, and whether modification may be optimal in early-life (aged <45 years), midlife (aged 45–65 years) or late-life (aged >65 years). Finally, barriers that could influence the ability of an individual to engage in risk factor modification and, ultimately, dementia risk reduction are discussed.

## Introduction

**C**linicopathological and epidemiological studies have identified a wide range of dementia risk factors on the basis of their association with cognitive decline as well as increased risk for cognitive impairment and dementia onset (1–7). The most unequivocal risk factors for dementia are biologically determined, such as older age, with 1 in 10 people over 65 but 3 in 10 people over 85 living with dementia (1). The Apolipoprotein E (*APOE*) ε4 allele is the strongest genetic risk factor for dementia caused by sporadic Alzheimer’s disease (AD), and is present in 9–23% of various ethnic populations (8). The risk of developing AD by 85 years of age is 18.4% for *APOE* ε4 heterozygotes and 48.3% for *APOE* ε4 homozygotes (9). Sex has also been identified as a risk factor, with more women developing AD dementia than men (1). While these risk factors cannot be modified to reduce dementia risk, there is now consensus that many dementia risk factors can be modified. For example, based on a large-scale review of observational studies, the Lancet Commission of Dementia Prevention, Intervention and Care estimated that approximately 40% of cases of clinically classified dementia could be attributed to twelve key modifiable dementia risk factors (MDRFs) (10). This supports that dementia has a multifactorial etiology and raises the possibility that many cases of dementia could be prevented by strategies that act to reduce MDRFs (11, 12).

MDRFs are increasingly the targets of dementia risk reduction and prevention strategies, many of which are now underway. For example, several randomized controlled trials (RCT) have been conducted to determine whether single- or multi-domain interventions that target subsets of MDRFs may delay or even prevent cognitive decline or onset of dementia (13), such as the global multi-domain Finnish Geriatric Intervention Study to Prevent Cognitive Impairment and Disability (FINGER) trial (14). In addition, dementia prevention guidelines have been published by the World Health Organisation (WHO) that outline recommendations of strategies for reducing dementia (15). Recently, a brain health service model to facilitate dementia risk detection and interventions in at-risk but cognitively unimpaired individuals has been introduced and proposed as a potential method for implementation of dementia risk reduction strategies into clinical practice (16).

While there is growing consensus regarding which dementia risk factors are modifiable, little consideration has been given to the extent to which individuals could identify whether they have one or more MDRFs, and if they do, how easy such factors are to modify. For example, for an individual seeking to reduce their dementia risk, an MDRF such as physical inactivity may be easier to identify and modify compared to air pollution. Relatedly, the time required for any modification of an MDRF to influence and then maintain any meaningful reduction in dementia risk is also likely to differ across MDRFs. Thus, characteristics of MDRFs such as ease of identification and targeting, and the period of modification required for reduction of dementia risk will each influence the extent to which strategies designed to minimize MDRFs could be implemented. This also becomes important for planning RCTs, where time intervals are often fixed and limited to less than five years (11, 17, 18). To date, factors that influence the feasibility of implementing behavior change strategies for dementia risk reduction or prevention have not been considered formally or conceptualized in the observational studies that have informed the design of dementia risk reduction strategies. Thus, despite widespread agreement that targeting MDRFs is important for dementia prevention, the guidelines and policies for brain health that are based on these studies do not contain information on the feasibility, or time interval necessary, for MDRF modification approaches. For example, in the Lancet Commission Report, a range of recommended strategies were simply listed for targeting MDRFs at an individual or population-based level (10). Further, in a recent report that considered the societal and equity challenges of a proposed brain health service model, it was acknowledged that for some individuals, accessing a brain health service may not be feasible due to disadvantage and that population-based approaches may be more appropriate for equitable dementia prevention (19). Whilst these reports introduced the premise of interventions that target MDRFs needing to occur at different levels, no definition or characterization of these different levels were provided which inhibits understanding of the feasibility of how these recommended strategies for dementia risk reduction can be implemented. As a foundational step to strengthen these and future recommendations for dementia risk reduction from research studies, the degree of modifiability of key MDRFs requires more formal conceptualization, which will ultimately increase the feasibility of achieving dementia risk reduction and prevention.

The primary aim of this review is to develop a framework for classifying the degree of modifiability of MDRFs for research studies. This framework interprets the modifiability of MDRFs from the perspective of an individual seeking to reduce their dementia risk and considers non-modifiable dementia risk factors, such as older age, the *APOE* ε4 allele and sex, as a basis for comparison or contextualization of MDRFs. Each dementia risk factor is classified as one of the following: (i) non-modifiable; (ii) modifiable with intervention at societal or community level; or (iii) modifiable with intervention at the individual level. This framework is applied to a review of well-established MDRFs, such as those identified in the Lancet Commission Report (10), as well as other MDRFs for which there is accumulating evidence for their association with increased risk for cognitive impairment, cognitive decline and dementia onset. The extent to which these MDRFs could be modified by an individual seeking to reduce dementia risk is determined, as well as the resources that might be needed for both MDRF identification and modification, and whether modification would be ideal in early-life (aged <45 years), midlife (aged 45–65 years) and late-life (aged >65 years) (10). Finally, this review considers some of the barriers and enablers that influence the feasibility of modifying MDRFs.

## Framework of modifiability for dementia risk factors

The framework for the degree of modifiability of dementia risk factors is summarized in Table 1. In this framework, each MDRF is classified according to its potential for modification by an individual seeking to reduce their dementia risk. This can occur in formal intervention trials or in everyday life. Three levels were developed for classifying the modifiability of each dementia risk factor which include:

(i) Non-modifiable – dementia risk factors that cannot be modified to reduce dementia risk.
(ii) Modifiable with intervention at the societal or community level – dementia risk factors that could potentially be modified but which require interventions from agencies, governments, or communities (e.g., government policy, societal change).
(iii) Modifiable with intervention at the individual level – dementia risk factors that can be modified by the individual with and/or without professional/medical interventions.

**Table 1:** Framework of degree of modifiability of dementia risk factors

**Level of modifiability**	**Risk factor**	**Identification**	**Modification**	**Ideal life stage of modification**
Non-modifiable				
	Older age	Date of birth, self-report	-	-
	APOE ε4	Genetic testing	-	-
	Female sex	Self-report or genetic testing	-	-
Modifiable with intervention at societal or community level				
	Low educational attainment	Self-report or school records	Ensure completing of primary and secondary schooling	Early-life ↑ (145)
	Low occupational complexity	Self-report or occupation classification codes applied to census data	Adopt more complex/challenging tasks at an individual’s current job or via career change to a more complex role	Early-life ↑ (145)
	Low SES	Self-report or external inspection of SES factors	Improve opportunity for educational attainment, occupational attainment, home ownership, and/or personal and family wealth through government and/or societal intervention or policy change	Early-life ↑ (146)
	Air pollution	Measurement of air pollutants by government or environmental agencies	Reduce pollutant emissions through large-scale government and societal intervention or policy change	Early-life ↑ (147)
	TBI	Medical examination and diagnosis	Public health initiatives such as policy change or TBI prevention programs implemented by sports programs, schools or governments	Early-life ↑
Modifiable with intervention at individual level				
	Hypertension	Blood pressure reading	Prescription of medication and/or lifestyle modification	Midlife (148)
	Hypercholesterolemia	Blood test to analyse plasma cholesterol levels	Prescription of medication and/or lifestyle modification	Midlife (149)
	Diabetes Mellitus	Blood test for glycaemic level analysis	Prescription of medication and/or lifestyle modification	Midlife (150)
	Obesity	Height and weight measurement to calculate BMI	Lifestyle modification with and/or without intervention from allied health or medical professionals; or prescription of pharmaceutical or surgical intervention	Early-life ↑ (151)
	Physical inactivity	Self-report or activity measures (e.g., smartwatches)	Lifestyle modification with and/or without professional intervention	Early-life ↑
	Poor diet	Self-report	Lifestyle modification with and/or without professional intervention	Early-life ↑
	Cigarette smoking	Self-report	Lifestyle modification with and/or without medical or professional intervention	Early-life ↑
	Excessive alcohol intake	Self-report	Lifestyle modification with and/or without medical or professional intervention	Early-life/Midlife (152)
	Depressive symptoms	Self-report and psychological assessment	Psychological and/or pharmaceutical treatment	Early-life/midlife(153)
	Anxiety symptoms	Self-report and psychological assessment	Psychological and/or pharmaceutical treatment	Early-life/midlife (154)
	Psychological stress	Self-report and/or measurement of cortisol levels	Psychological treatment and/or other professional intervention (i.e., mindfulness programs)	Early-life/midlife (88)
	Low cognitive engagement	Self-report	Lifestyle modification with and/or without professional intervention	Early-life/midlife (155)
	Social isolation	Self-report	Lifestyle modification with and/or without professional intervention	Early-life/midlife (156)
	Short sleep duration	Self-report or sleep assessment	Psychological and/or pharmaceutical treatment, and/or improvement of sleep hygiene	Early-life/midlife (27)
	Excessive sleep duration	Self-report or sleep assessment	Psychological and/or pharmaceutical treatment, and/or lifestyle modification	Early-life/midlife (157)
	OSA	Sleep assessment	Prescription of medical intervention (e.g., CPAP)	Midlife
	Hearing loss	Self-report and hearing assessment	Prescription of medical intervention (e.g., hearing aids, cochlear implants) and/or lifestyle modification to increase cognitive/social engagement	Midlife (10)
	Vision impairment	Self-report and eye assessment	Prescription of medical intervention (e.g., vision corrections such as glasses or laser eye surgery) and/or functional aids and/or lifestyle modification to increase cognitive/social engagement	Early-life ↑

Note. References in the ideal life stage of modification column represent empirical evidence supporting the proposed life stage for the MDRF. If there is no reference, then the proposed life stage is hypothetical. APOE ε4=Apolipoprotein E ε4 allele; SES=socioeconomic status; TBI=traumatic brain injury; OSA=obstructive sleep apnea; BMI=body mass index; CPAP= continuous positive airway pressure; Early life ↑=ideal to modify early life and onwards.

For each MDRF, the classification was made by characterizing information on how an MDRF can be modified, the resources required for modification, and the potential for modification by the individual. Whilst this framework does not offer quantification for the levels of modifiability, it introduces the concept and forms the basis for this to be developed in future studies. The following section reviews each MDRF and provides the theoretical basis for its classification within the modifiability framework.

Substantial evidence from observational studies, synthesized in the Lancet Commission Report,10 has identified low education, hearing loss, traumatic brain injury (TBI), hypertension, excessive alcohol intake, obesity, smoking, depression, social isolation, physical inactivity, air pollution, and diabetes as well-established MDRFs (10). Other MDRFs, that were not highlighted by the Lancet Commission Report but for which there is a growing evidence-base from epidemiological and clinicopathological studies include low occupational complexity (20), hypercholesterolemia (21), anxiety (22), psychological stress (23), low socioeconomic status (24), poor diet (25), low cognitive engagement (26), short sleep duration (27, 28), excessive sleep duration (29), obstructive sleep apnea (OSA) (30) and vision impairment (31). These MDRFs are therefore also included in the framework to ensure it is as comprehensive as possible. Definitions and operationalization for each MDRF are summarized in Table 2.

**Table 2:** Definition and operationalization of modifiable dementia risk factors

**MDRF**	**Definition and operationalization**
Low educational attainment	Not completing primary and/or secondary school, or having less than twelve years of formal education (10)
Low occupational complexity	Low complexity of job tasks, often in different categories (i.e., working with people, data, things) (20); no established cut-off
Low SES	Social and economic determinants of an individual or group’s position within society, operationalized typically using low household income as a proxy or a low composite score based on multiple factors such as educational attainment, occupational role, home ownership, family income and family wealth (24, 158); no established cut-off
Air pollution	Contamination of the environment by a substance (e.g., chemical) that modifies natural elements of the atmosphere, operationalized typically as exposure to specific pollutants such as particulate matter (PM), sulfur dioxide (SO2) and carbon monoxide (CO) (159, 160)
TBI	Acute brain injury resulting from head trauma, operationalized typically using medical diagnosis or self-report (161)
Hypertension	Persistent elevated blood pressure, operationalized as systolic blood pressure of 130 mmHg or greater or diastolic blood pressure of 80 mmHg or greater (148, 162)
Hypercholesterolemia	Cardiovascular condition characterized by elevated plasma total cholesterol level, operationalized as reaching a threshold such as 6.5 mmol/L (163)
Diabetes Mellitus	Collective term for various metabolic conditions that are characterized by chronic hyperglycemia, or high blood glucose, operationalized typically through blood glycemic levels of 7.0 mmol/L or greater (164, 165)
Obesity	Condition characterized by excessive body fat that is associated with increased risk of health complications, operationalised typically as BMI of 30 or greater (166, 167)
Physical inactivity	Lack of regular movement or exercise, operationalized typically as insufficient to meet established physical activity guidelines (e.g., 150 minutes of physical activity per week) (168)
Poor diet	Overconsumption of foods that are considered unhealthy, inflammatory or have low nutrition (e.g., highly processed, high sugar) and underconsumption of foods that are nutritious or beneficial to health (e.g., fruits and vegetables), operationalized typically as high adherence to a Western diet (i.e., high fat, sodium, sugar and processed food) or inflammatory diet (i.e., high intake of processed meats, refined carbohydrates, soft drinks), low intake of fruit and vegetables or low adherence to established dietary guidelines or an evidence-based neuroprotective diet (i.e., Mediterranean or MIND diet) (74, 75, 169)
Cigarette smoking	Act of inhaling and exhaling the fumes of burning tobacco in cigarette form, operationalized typically by current smoking status (170)
Excessive alcohol intake	Regular alcohol consumption at a level that is determined to be harmful to one’s health, operationalized typically as amount of alcohol units or frequency/number of alcoholic beverages during a given period of time that is greater than established alcohol safety guidelines (e.g., more than 10 standard drinks per week, &gt;21 units per week) (10, 171, 172)
Depressive symptoms	Experience or report of symptoms that typically characterize depression, operationalized typically by meeting or surpassing established clinical thresholds on scales/questionnaires (173)
Anxiety symptoms	Experience or report of symptoms that typically characterize anxiety, operationalized typically by meeting or surpassing established clinical thresholds on scales/questionnaires (174)
Psychological stress	Experience of an individual when they perceive that the demands of an environmental challenge exceeds their ability to adapt or cope, operationalized typically as perceived stress measured using validated scales with clinical thresholds, self-reported experience of stressful life events, or stress vulnerability including personality traits such as neuroticism (23, 175, 176)
Low cognitive engagement	Lack of or limited engagement in activities that are cognitively stimulating, operationalized typically as the frequency or variety of engagement in cognitively stimulating leisure activities (e.g., playing a musical instrument, artistic activities); no established cut-off (177, 178)
Social isolation	Lack of or limited engagement in socially stimulating interactions or activities, operationalized typically as frequency of social engagement/contact (i.e., seeing friends and/or family) or by estimating social network size; no established cut-off (179)
Short sleep duration	Average number of hours spent asleep per night which is less than established guidelines for healthy or beneficial sleep, operationalized typically as six or less hours of sleep per night (27)
Excessive sleep duration	Average number of hours spent asleep per night which is more than established guidelines for healthy or beneficial sleep, operationalized typically as eight or more hours of sleep per night (27)
OSA	Ongoing events of upper airway obstruction, fragmented sleep and hypoxia during sleep, operationalized typically by medical diagnosis (180, 181)
Hearing loss	Decreased sensitivity to sounds and impaired speech perception initially affecting higher frequencies/pitches and is considered to develop due to idiopathic degeneration of inner ear structures, operationalized typically as self-reported symptoms or diagnosis (182)
Vision impairment	Reduced visual acuity that without correction (i.e., via glasses) interferes with daily function and can be a result of several causes such as cataracts or macular degeneration, operationalized typically as self-reported symptoms or diagnosis (183)

Note. SES=socioeconomic status; TBI=traumatic brain injury; BMI=body mass index; MIND= Mediterranean-DASH Intervention for Neurodegenerative Delay; OSA=obstructive sleep apnea.

### Search criteria

PubMed and Google Scholar databases were searched for articles published in English with no date or time restrictions. There were several steps to the literature search process for the current review. First, to obtain evidence of an association between a given MDRF with cognitive decline and risk of cognitive impairment and/ or dementia, a combination of search terms was included such as “modifiable dementia risk factor”, or the specific MDRF (e.g., “hypertension”) and “cognitive decline”, “cognitive impairment”, or “dementia risk”. To determine from previous literature the methods for identification of MDRFs, a term for the specific MDRF was searched for in combination with terms relating to identification such as “detection”, “measurement”, “identification”. To determine from previous literature strategies for MDRF modification or intervention, combinations of search terms then included the specific MDRF and terms for modification such as “intervention”, “modification”, “treatment”. In some cases, this process was guided by intuition and prior knowledge as for some MDRFs such specific details have not been explicitly defined in previous studies. To propose ideal life stages of modification for MDRFs, search terms included the specific MDRF, “dementia risk” and “age”, or “early life”, “midlife”, or “late life”, or “middle-aged adults”, or “older adults”. Finally, to search previous literature for barriers and enablers to dementia risk reduction, search terms included “dementia risk reduction”, “dementia prevention”, or “dementia risk”, and “barrier” or “enablers” and “engagement”, “intervention”, or “behavior”. Terms such as “external”, “population-based”, “internal” or “individual” were also subsequently added to the literature search for dementia risk reduction barriers.

## Dementia risk factors that are modifiable with intervention at societal or community level

Table 1 shows several MDRFs that are classified as modifiable with societal or community level intervention. The modification of these MDRFs is difficult or not feasible for individuals to achieve themselves. Rather, for modification of these MDRFs, interventions from agencies, governments, or communities (e.g., government policy, societal change) may be required.

### Low educational attainment

Low educational attainment, or less education, can be identified in research studies or in the general population by self-report or via inspection of school records. Low educational attainment may be modified in early-life by ensuring completion of at least primary and secondary schooling (10), however, the evidence is equivocal on whether additional education in later life stages is associated with reduced dementia risk (32). Importantly, low educational attainment is related to low socioeconomic status (SES) (33), which can create barriers to health literacy, healthcare access and opportunity to engage in health-promoting behaviors. Thus, increasing educational attainment in early life is facilitated by factors external to the individual such as parents or guardians (e.g., ensuring completion of secondary school), or at a government or societal level (e.g., free, or subsidized education, education promotion campaigns, universal access to education).

### Low occupational complexity

Low occupational complexity can be identified by self-report or, in some research studies, by occupational classification codes applied to census data (34). Low occupational complexity may be modified by adopting more complex or challenging tasks at an individual’s current job or via career change to a more complex role or industry. However, the ability to make such modifications may differ according to occupation type and industry, and in many cases, may rely on opportunities for further education or upskilling. Importantly, this may not be possible for individuals seeking to reduce their dementia risk due to challenges such as financial responsibilities and caregiving. Thus, modification of occupational complexity may need to occur in early adulthood, the typical time of occupational training and career/work initiation. However, opportunity to increase occupational complexity may also be related to SES (35) and may require intervention from factors external to the individual (e.g., workplace, government/policy, and even parents or guardians, given its relation to early-life educational attainment). Further, opportunity to increase occupational complexity can also depend on whether free or subsidized tertiary education is available where the individual lives.

### Low SES

Low SES can be identified via self-report or external inspection of socioeconomic factors including educational attainment, occupational role, home ownership, family income and family wealth. Modification of low SES may not be in the control of the individual. Low SES likely relates to multiple converging factors and social determinants of health such as barriers to health literacy (36), limited access to healthcare and community resources that promote healthy behaviours (e.g., green spaces, affordable fresh food) (37), as well as structural factors, such as social exclusion, discrimination, and racism (38, 39). These factors may affect individuals throughout the lifespan, and may work independently or in synergy to influence engagement in risky lifestyle behaviors that often promote morbidity such as cardiovascular conditions and dementia (40). SES may therefore be difficult and complex to modify at the individual level and likely requires larger scale government and societal intervention or policy change.

### Air pollution

Air pollution can be identified by the measurement of air pollutants such as particulate matter (PM), sulfur dioxide (SO2) and carbon monoxide (CO) by government bodies or environmental agencies (41, 42). Air pollution may possibly be modified by targeting known contributors (41, 43) at the individual-level (e.g., smoking cessation, the adoption of electric cars and air purifiers). However, this can require significant financial and personal resources, and it remains unclear whether this will result in positive short-term health changes for the individual. Further, it may not be feasible for individuals to relocate from an area or city with high air pollution to another with lower pollution to reduce their dementia risk. Given this, strategies to reduce dementia risk from air pollution are more likely to occur through modification at a governmental and/or large-scale societal level (44). The Lancet Commission Report identified air pollution as a late-life MDRF (10), however, it is unclear whether this reflects lifetime exposure to air pollution or whether air pollutants exacerbate neurodegenerative changes already occurring in late life (45).

### Traumatic brain injury

TBI can be identified by medical examination and diagnosis or by self-report diagnosis by the individual after the event. If an individual has already experienced TBI, some treatments can be administered to individuals by medical practitioners to reduce the severity of damage to the brain (46), although it is unclear whether these treatments will then, in turn, reduce dementia risk. Thus, once an individual has already experienced TBI, the risk for dementia posed by TBI may not be possible to modify or reverse at the individual level. Contact sports and motor vehicle accidents both have high incidence rates of TBI (47, 48). Thus, it may be more practical to modify TBI associated risk for dementia through public health initiatives such as targeted preventative educational programs and policy changes that are external to the individual and focused on protecting the brain from injury (i.e., mandatory helmets for football players, safer roads/cars). This may occur either at the government level, by schools and sport clubs, or by raising public awareness of the guidelines and recommendations around TBI risk. The Lancet Commission Report identified TBI as a midlife MDRF likely due to the amount and quality of evidence that exists for this life stage (10). It is unclear whether experiencing TBI in childhood or adolescence (e.g., though playing sport) is also associated with dementia risk in later life, although there is some evidence that TBI-related dementia risk is attenuated with time (49).

### Summary

The MDRFs considered so far have been classified as modifiable with intervention at societal or community level, such as government policy or even legislative change. Some interventions required to modify these MDRFs are preventative, such as educational programs and policy changes for preventing TBI, whilst others are likely ameliorative such as government policy to reduce emission of air pollutants. Many large-scale societal and community level interventions that require policy change take time, effort and resources and therefore may not be entirely feasible for meaningful dementia risk reduction. Further, the efficacy of these population-level approaches to dementia risk reduction is currently unclear as they have been severely under-researched (50). Additionally, most of these MDRFs require intervention in early life. Whilst some interventions may already occur early in life such as completion of schooling or educational programs to prevent TBI (i.e., in sports programs) for reasons other than dementia prevention (i.e., general child wellbeing or health), it is unclear the extent to which these interventions directly or indirectly influence dementia risk and whether public health initiatives specific for dementia prevention in children, adolescents or young adults are plausible.

## Dementia risk factors that are modifiable with intervention at the individual level

Table 1 summarizes MDRFs classified as being modifiable with intervention by the individual. Depending on the individual, MDRF and specific circumstances, this may occur with or without the individual accessing medical or other professional intervention.

### Hypertension and hypercholesterolemia

Hypertension can be identified through blood pressure monitoring which is a simple and non-invasive assessment that allied health professionals and medical practitioners can administer (51). Similarly, hypercholesterolemia can be identified by simple blood tests to monitor plasma cholesterol which can increase opportunity for early detection (52). Hypertension and hypercholesterolemia can both be modified at the individual level through the prescription of pharmaceutical treatment (e.g., benazepril, statins) (53, 54) and/or lifestyle modification (e.g., diet or exercise programs) (55, 56), by medical practitioners. The relationship between hypertension and hypercholesterolemia with increased dementia risk is strongest when these conditions present in midlife (57, 58), with some positing that chronicity (i.e., the condition is present for a longer timespan compared to if present from late-life only) may underly this observation (59). Accordingly, midlife is considered the ideal age for modification of these MDRFs (10, 58).

### Diabetes Mellitus

Diabetes mellitus (DM) can be identified by blood tests for glycemic level and medical diagnosis. Blood tests are recommended for people at greater risk, including middle-aged adults, those with obesity, or a those with a family history of DM, which can facilitate early detection and treatment (60). DM can be modified by the individual through medications (e.g., metformin) and lifestyle modification such as limiting sugar intake and increased physical activity (61) prescribed by medical practitioners. Whilst DM has generally been considered a late-life MDRF, however, DM management is recommended to occur as early as possible (10, 62).

### Obesity

Obesity can be identified via simple height and weight measurement to calculate BMI which can be done by individuals themselves or administered by allied health professionals/medical practitioners. Obesity can be addressed by individuals through lifestyle modification (e.g., increase in exercise, diet modification) (63, 64), which can be supported by allied health professionals (e.g., personal trainer, dietician, exercise physiologist), or pharmacological/surgical interventions (e.g., appetite suppressors, bariatric surgeries) (65). Obesity is most consistently associated with dementia risk outcomes when present in midlife (66), suggesting that midlife is an ideal timeframe for modification. However, given the strong link between obesity and other morbidities such as cardiovascular disease (67), it would be prudent for obesity to be addressed as early as possible.

### Physical inactivity

Physical inactivity, or sedentary behavior, can be identified by self-report by the individual or through wearable measures of activity (e.g., smartwatches). Physical inactivity can be modified by individuals through lifestyle modification to increase physical activity, and can be supported by professional resources such as personal training, gym membership, and councilrun community fitness programs (68). Community resources such as access to parks, walking trails, and bicycle paths can also promote engagement in physical activity (69). Whilst the Lancet Commission Report identified physical inactivity as a late-life MDRF (10), the relationship between physical inactivity and dementia risk in older adults is complex and may also contain reverse causation, i.e., sedentary behavior or physical inactivity in older adults may be indicative of underlying neurodegeneration, frailty, and incipient dementia as opposed to physical inactivity acting as a risk factor for dementia. Given its relationship with obesity, and the substantial benefits that physical activity has on improving cardiovascular health (70), it is therefore prudent for physical inactivity to be addressed at earlier life stages.

### Cigarette smoking

Cigarette smoking is identified via self-report by the individual. Cigarette smoking can be reduced or ceased by individuals through lifestyle modification, however, nicotine addiction that is commonly associated with long-term smoking is a barrier to smoking reduction/cessation for many individuals. Smoking reduction/cessation can be aided with medical support, pharmaceutical aids (i.e., nicotine patches, medication), support hotlines and counselling (71). The Lancet Commission Report identified cigarette smoking as a late-life MDRF (10). However, the effects of cigarette smoking on dementia risk are likely a result of chronic or lifetime exposure, and may be confounded by survival bias given the greater risk of premature death associated with smoking (72). Importantly, general medical advice recommends strongly that smoking reduction or cessation occurs as early as possible (73).

### Poor diet

Poor diet is identified by self-report of dietary patterns by individuals. Diet can be modified by individuals through lifestyle modification to reduce intake of unhealthy or inflammatory foods (74) and increase adherence to dietary guidelines or neuroprotective diets (e.g., Mediterranean-DASH Intervention for Neurodegenerative Delay diet) (75). Individuals can also be supported through consultation with dieticians or nutritionists, or access to meal plans/recipes. Poor diet is related to increased inflammation (76) and health conditions such as cardiovascular conditions (e.g., hypercholesterolemia) (77) which are also associated with increased dementia risk. Thus, given the robust evidence for associations between neuroinflammation and midlife cardiovascular conditions with dementia risk, it may be prudent for poor diet to be targeted at midlife or an earlier life stage.

### Excessive alcohol intake

Excessive alcohol intake can be identified by self-report of alcohol intake by individuals, which can be assessed as excessive by medical or health professionals using established alcohol safety guidelines (e.g., no more than 10 standard drinks per week), or via self-reported concerns by individuals themselves, their family and/ or friends. Excessive alcohol intake can be reduced by individuals through lifestyle modification. However, alcohol addiction and dependence can act as significant barriers to reduction (78), and medical or psychological intervention may be required for individuals that experience alcohol addiction/dependence. Whilst midlife is generally considered to be the ideal stage to reduce alcohol intake (10), general medical practice recommends limiting excessive alcohol intake as early as possible (79).

### Elevated depression and anxiety symptoms

Elevated depression and anxiety symptoms can be identified by self-report of symptoms by individuals which can then lead to psychological assessment. Depression and anxiety symptomatology can be reduced using psychological (e.g., cognitive behavioral therapy) (80) or pharmaceutical (e.g., anti-depressant medication) (81) interventions administered to individuals by psychiatrists, general practitioners, psychologists and counsellors. Other interventions can complement pharmaceutical or psychological therapy such as physical activity (82) and mindfulness practice (83) which individuals can engage in with assistance from professional programs, online resources, or digital application-based interventions. The Lancet Commission Report identified depression as a late-life MDRF (10), however, anxiety or depression in late-life are also symptoms of incipient dementia (84, 85). Therefore, risk for dementia that is contributed by depression and anxiety symptomatology may need to be targeted at earlier life stages.

### Psychological stress

Psychological stress can be identified by self-report experience of individuals or measurement of cortisol levels in blood, saliva or hair samples (86). Psychological stress may be addressed by individuals via psychological treatment which can be administered by psychiatrists, psychologists, or counsellors. Complementary to psychological treatment, mindfulness practice has also been associated with improvements in psychological stress which individuals can access via professional programs and online resources (87). Several studies show that midlife is typically stressful due to career demands and caregiving responsibilities, and this heightened psychological stress is associated with increased dementia risk several decades later (88). Therefore, although reducing psychological stress is recommended at any life stage, midlife is an important time to implement strategies to cope with stress.

### Low cognitive engagement and social isolation

Low cognitive engagement and social isolation can be identified by self-report of engagement in cognitively and socially stimulating activities or interactions by individuals. Attempts to increase cognitive and social engagement (e.g., engagement in creative and/or social activities) can be made by individuals, and can be facilitated by professional programs (e.g., community groups, piano lessons) in the community and/or online. Social isolation was identified as a late-life MDRF in the Lancet Commission Report (10), however, social isolation in late-life may also reflect withdrawal from usual activities associated with incipient dementia (89). It is possible that any neurological benefits of cognitive and social engagement (i.e., cognitive reserve) (90) occur as a lifelong process, suggesting that low cognitive engagement and social isolation should be addressed as early as possible.

### Sleep-related characteristics and disorders

Short (≤6 hours per night) or excessive (≥8 hours per night) sleep duration can be identified by self-report and in some cases, by consumer grade devices such as smartwatches or polysomnography (i.e., in research studies, sleep clinic). Short sleep duration can be addressed and increased to the optimal seven hours with interventions such as pharmaceutical treatment (e.g., melatonin) (91), or improvement of sleep hygiene (92). Excessive sleep duration can also be addressed by pharmaceutical treatment (e.g., wakefulness promoting agents or stimulants) and lifestyle modification (e.g., alarm use, caffeine), however, is often associated with underlying conditions which themselves require treatment (93). Further, as both short and excessive sleep duration are related to depressive symptoms (29), psychological treatment or practices may be required for some individuals (94). These interventions can be administered to individuals by medical practitioners, sleep specialists, psychologists or can be accessed through community and online resources (e.g., sleep hygiene information, mindfulness classes, digital application-based interventions). While short sleep is very common in late-life and has also been proposed to be a symptom of incipient dementia (95), short sleep in midlife is also associated with increased dementia risk (27) indicating that intervention should occur in midlife or earlier life stages. While the relationship between excessive sleep duration and risk of dementia in midlife or earlier is unclear (27) and late life excessive sleep is also proposed as a symptom of incipient dementia (96) intervention should likely also occur as early as possible.

OSA can be identified by medical diagnosis following sleep analysis using polysomnography. OSA can be treated through prescription of interventions for OSA to individuals by medical practitioners, such as continuous positive airway pressure (CPAP) (97), which has shown promise in attenuating the negative consequences of OSA on cognition and the brain (98). OSA that presents at any time during adulthood, but particularly from midlife, has been shown to have deleterious effects on the brain (99) and thus requires modification as early as possible once diagnosed.

### Sensory impairments

Hearing loss and vision impairment can be identified by self-reported symptoms by the individual and/or assessment by health professionals (i.e., audiologist, optometrist). Age-related hearing loss can be addressed by individuals through the prescription of interventions such as hearing aids or cochlear implants by medical practitioners and audiologists, with some studies showing improvements in cognition and diminished association with cognitive decline associated with these interventions (100). Vision impairment can be addressed by individuals through prescription of corrective interventions such as glasses, contact lenses, or laser eye surgery (101), or functional aids such as visual aid technologies and rehabilitation (102). For instance, one study showed that individuals with cataracts that had corrective surgery had a 50% reduced risk of dementia compared to those with cataracts who did not have surgery (31). One hypothesis for how hearing loss and vision impairment may increase risk for dementia is through diminished cognitive and social engagement and depressive symptoms, which themselves are risk factors for dementia (31, 103). For instance, reduction in social isolation, loneliness and depressed mood partially mediated the association between hearing aid use and risk of dementia (103). Age-related hearing loss begins to emerge in midlife (104), thus making midlife the ideal timeframe for intervention (10). Vision impairment can impact an individual at any age so is recommended to be addressed or corrected as early as possible.

### Summary

Classifying MDRFs according to this framework of modifiability highlights the issue that showing associations between MDRFs and cognitive decline or dementia may not necessarily translate readily to simple and easy MDRF identification, modification and dementia risk reduction for individuals. Therefore, the characterization of the feasibility of identification and modification of MDRFs, and the resources needed, contributes to enhancing understanding of how meaningful dementia risk reduction can be achieved. Thus, implementing a framework such as this to current and future studies of MDRFs forms a basis for reducing heterogeneity in the field, increasing general understanding about dementia risk reduction, and optimizing risk reduction strategies that target MDRFs.

## Ideal life-stage for MDRF modification

As is illustrated in Table 1, most MDRFs were classified as being modifiable with intervention at the individual level which indicates considerable opportunity and feasibility for dementia risk reduction. Table 1 also shows that the ideal life stage proposed for modification of all MDRFs is early-to-mid-life and onwards. This is in contrast to the Lancet Commission Report which indicated that six MDRFs were classified as late-life MDRFs (10). However, these six late life MDRFs, such as physical inactivity, social isolation and depression, might be indicators of withdrawal or symptoms attributable to incipient dementia and thus indicative of reverse causation. Importantly, underlying AD pathological processes can develop over decades before clinically recognizable symptoms are detectable, even in middle-aged adults (105, 106). Therefore, although the life-course model of dementia risk proposed by the Lancet Commission Report suggests that some MDRFs have the greatest influence on dementia risk in late life, it is possible that, for some individuals, this may be too late for dementia risk reduction to be achieved. Additionally, patterns of health and lifestyle behaviors likely develop from an early age (107), which may mean that the influence of some MDRFs accumulate across the lifespan and meaningful behavior change and dementia risk reduction in later life can be difficult. Further, many MDRFs (e.g., smoking, physical inactivity) are also risk factors for other diseases and negative health consequences, such as cardiovascular disease, which may emerge prior to dementia. The current framework thus proposes that if MDRFs are present earlier in life, it would be prudent for modification to occur as early as possible. However, it is important to acknowledge that several factors, internal and external to the individual, may contribute to the extent to which an individual can engage in dementia risk reduction behavior even if their MDRFs are readily modifiable. The following sections discuss barriers and enablers to dementia risk reduction behavior.

## Barriers and enablers to dementia risk reduction

**Figure 1:**
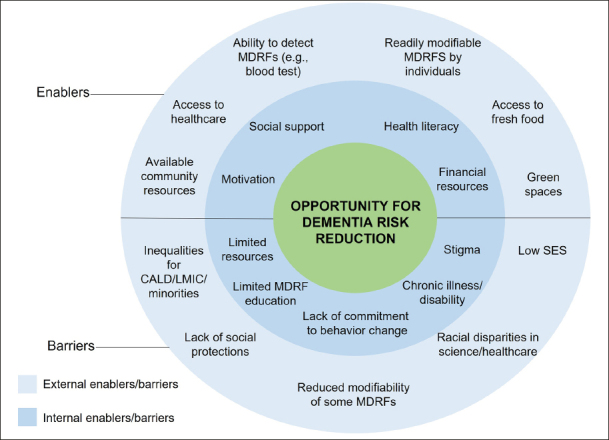
External and internal barriers and enablers that influence opportunity for individuals to engage in dementia risk reduction behavior Note. MDRF=modifiable dementia risk factor; SES=socioeconomic status; CALD=Culturally and Linguistically Diverse; LMIC=low-middle income countries.

Dementia risk factors that are modifiable by the individual have a higher level of modifiability as they can be targeted on an individual basis. Individuals can make major contributions to their own health and wellbeing in their everyday lives through adopting health-promoting behaviors (e.g., physical activity) and avoiding health-compromising behaviors (e.g., smoking), with or without medical or professional intervention (108). Further, by seeking assistance from medical or other professional interventions, individuals can take steps to improve health conditions (e.g., hypertension, OSA). However, even if some MDRFs are more feasible to target on an individual level, modification of these MDRFs may not necessarily occur or be easy to achieve. Both internal and external barriers can negatively influence engagement and maintenance of health behavior change relating to dementia risk. Alternatively, there are also internal and external enablers that can promote or facilitate engagement in dementia risk reduction behavior. These are summarized below and synthesized in Figure 1.

### Internal barriers to dementia risk reduction

A key internal, or individual-based, barrier to dementia risk reduction is an individual’s knowledge and beliefs surrounding MDRFs. Large-scale surveys suggest that many in the general population are unaware of MDRFs and have relatively low dementia literacy (109, 110) which may lead to non-evidence-based beliefs and attitudes (e.g., dementia is a normal part of ageing) (111) and limit opportunity for MDRF identification which, for many MDRFs, relies on self-assessment. The limited knowledge of dementia and its risk factors may also contribute to internalized stigma and fear towards dementia, which may deter individuals from learning about and engaging in dementia risk reduction strategies (112–114). Individual barriers may also include limited resources such as lack of time or finances to dedicate towards dementia risk reduction strategies, limited support structures and diminished self-efficacy (i.e., individual’s belief in their abilities to accomplish a goal) (115). Chronic illness or difficulties with mobility may also act as barriers to targeting some MDRFs (i.e., physical inactivity) at the individual level (116). Lastly, motivation and commitment to long-term behavior change is essential to achieve and sustain dementia risk reduction, however, is difficult to maintain for many individuals (36, 117–119). Person-centred interventions that address these barriers will be essential to promoting long-term behaviour change, and, in turn, reducing dementia risk (18, 120).

### Internal enablers to dementia risk reduction

Some enablers that are internal to the individual may increase the likelihood of engaging in and maintaining dementia risk reduction behavior, such as autonomous motivation (i.e., engaging in behavior which is perceived as consistent with one’s goals) (121). For example, higher autonomous motivation was a key mediator of successful outcomes across a range of obesity-related lifestyle interventions (122). Further, having social support, as a key social determinant of health, may also increase motivation and engagement in certain health promoting behaviors, such as physical activity (123). Additionally, having personal financial resources to expend may also increase the likelihood of individuals reducing their MDRFs (e.g., joining a gym, engaging in psychological therapy). Finally, an important internal enabler is health literacy, which is an individual’s cognitive and social ability to consume and understand health promoting information (124). Higher health literacy is associated with increased engagement in health behaviors, such as physical activity and healthy diet, as well as engagement in social activities (125), each of which are associated with dementia risk. Thus, individuals with higher health literacy may be more likely to engage in and maintain dementia risk reduction behavior.

### External barriers to dementia risk reduction

While agency is important for individuals to modify and maintain healthy lifestyle patterns and behaviors, external and structural barriers can influence opportunity for engagement in dementia risk reduction behavior (126). These barriers include SES both at the personal (i.e., financial) and neighborhood level which influence access to healthcare, education, fresh and nutritional food and green spaces or facilities that promote physical activity and other healthy behaviors (24, 37, 127). Additionally, interventions for certain MDRFs can be expensive and not always accessible (e.g., CPAP machines, hearing aids, psychological treatment) (128, 129). Accessing these interventions can depend on the health care system and governmental support available within one’s country, and individuals’ ability to afford private health insurance. Further, ability to choose health promoting behaviors is dependent on basic social protections such as housing and welfare, which creates barriers for dementia risk reduction in homeless populations (130). Ability for individuals to choose health promoting behaviors is also dependent on the environment in which they live, which suggests that barriers to choosing health-promoting behaviors may increase with the increasing threat of the climate crisis (131).

The incidence of MDRFs will vary across countries and cultures, but many MDRFs cluster around inequalities – disproportionally affecting Culturally and Linguistically Diverse (CALD) populations and residents of low- or middle-income countries (LMICs) (132–134). Barriers to engaging in dementia risk reduction behaviors have in turn disproportionately affected these populations (135, 136). Most observational studies and intervention trials studying or targeting dementia risk typically recruit white, highly educated, and affluent participants. Whilst important insights are obtained from these studies, they are limited in their generalizability and do not account for these barriers (137, 138). This creates racial and socioeconomic disparities in dementia science and healthcare with potential to significantly interfere with dementia risk reduction practices for many individuals (139, 140). For example, whilst the current dementia risk reduction literature is replete with recommendations for the Mediterranean-DASH Intervention for Neurodegenerative Delay (MIND) diet, this recommendation may not be appropriate for individuals from CALD populations who are unfamiliar with or whose culture does not align with foods recommended in the MIND diet. Additionally, foods recommended in the MIND diet may not be accessible on a global level and can be highly dependent on where one lives. Greater efforts to address barriers to dementia risk reduction, particularly in CALD populations, racial minorities and LMICs, will be essential, as this remains an important but underrecognized aspect of the dementia risk reduction field.

### External enablers to dementia risk reduction

Many external enablers to dementia risk reduction relate to access to facilities or resources that promote healthy behaviors in the neighborhood (i.e., neighborhood advantage) (127) and/or country in which an individual lives. For example, having access to resources in the community such as gyms or libraries, access to fresh food in stores or supermarkets, and green spaces (e.g., parks in urban environments) (141) may increase the likelihood that individuals will engage in and sustain health promoting behaviors and dementia risk reduction strategies (142). Living in a country with universal healthcare may also increase access to medical interventions for many MDRFs including CPAP, medications for cardiovascular conditions, psychological treatment and hearing aids, particularly for those who may not be able to afford private health insurance (143). Further, access to healthcare would also increase detection of some MDRFs through more affordable and routine health checks such as blood pressure monitoring, blood tests for glucose and cholesterol, and hearing and eyesight checks.

Finally, the higher level of modifiability (i.e., modifiable at the individual level) of many MDRFs may also act as an external enabler as this increases feasibility of these MDRFs being targeted by individuals seeking to reduce their dementia risk.

## Conclusion

The current review extends previous reports such as the Lancet Commission Report by providing a formal framework to characterize the different levels of modifiability of MDRFs for application in studies aiming to inform dementia risk reduction and prevention strategies, such as intervention trials, guidelines and implementation into clinical practice. This framework also highlighted that the identification of MDRFs also requires different resources which can also influence engagement and achievement of MDRF modification and dementia risk reduction. MDRFs that are modifiable with intervention at the societal or community level require population-based strategies such as government policy change. MDRFs that are modifiable with intervention at the individual level have greater potential for reducing dementia risk and can be targeted more readily in intervention trials and in individuals’ daily lives. This review also proposes that the ideal life stage for intervention of most MDRFs by individuals seeking to reduce their dementia risk is midlife (e.g., aged 45–65 years) (10) or earlier. However, there remain important external (population-based) and internal (individual-based) barriers to reducing MDRFs. It has been proposed that a population-based approach is essential to achieve meaningful and equitable dementia risk reduction (144). It may take a village to prevent dementia. However, large-scale governmental and policy changes take time, effort, and resources to implement and may not address important internal barriers to dementia risk reduction (i.e., motivation and commitment for behavior change). It is thus essential to take both a population-based (144) and a person-centred (119) approach to ensuring that individuals are empowered, supported, and have the means, knowledge and tools necessary to reduce their readily modifiable dementia risk factors.
